# Do attachment styles influence the sexual function of an individual?

**DOI:** 10.1192/j.eurpsy.2024.1597

**Published:** 2024-08-27

**Authors:** J. C. Rodrigues, T. P. Soares, L. M. Ribeiro

**Affiliations:** Psychiatry, Vila Nova de Gaia/ Espinho Hospital Center, Vila Nova de Gaia, Portugal

## Abstract

**Introduction:**

Attachment theory, first proposed by John Bowlby and later extended by Mary Ainsworth and others, outlines how experiences of early childhood attachment with caregivers can affect one’s emotional and interpersonal relationships throughout adulthood. Typically, attachment styles are categorised into four main types: secure, anxious-ambivalent, avoidant and disorganised. Conversely, it is recognised that various biological, psychological, relational, social and iatrogenic factors elements can impact an individual’s sexual function.

**Objectives:**

Our aim with this research was to present the most current literature on whether there is a correlation between attachment styles and sexual function.

**Methods:**

We conducted a non-systematic review on the topic using PubMed and PsycInfo.

**Results:**

There is evidence indicating a link between attachment styles and sexual function.

People with secure attachment styles tend to experience more positive and fulfilling sexual relationships. Such individuals typically have a more positive self-image, they feel at ease with emotional intimacy, and are therefore able to openly communicate their needs and desires. They exhibit a healthy balance between seeking closeness and maintaining independence.

Individuals with anxious attachment styles may experience heightened levels of sexual anxiety and insecurity. Concerns regarding rejection or abandonment within sexual relationships may impact their sexual function and satisfaction. These individuals usually have a negative self-image and may be more prone to seek reassurance and validation through sexual activities.

People with avoidant attachment styles may encounter obstacles in developing emotional intimacy and closeness, which can negatively affect their sexual relationships. Such individuals might experience commitment anxiety and prioritise physical aspects of sexual activity over emotional bonding, ultimately decreasing sexual satisfaction for both them and their partners.

Disorganised attachment styles are linked with challenges in regulating their emotions and behaviours in intimate situations, which can have a negative impact on sexual function and satisfaction.

**Conclusions:**

While the literature proposes attachment styles may impact sexual function, it is important to acknowledge other factors that contribute to sexual function. In addition to biological and iatrogenic factors, individual personality, relationship dynamics, past experiences, and cultural influences all have a significant role in shaping one’s sexual behaviour and satisfaction. All of these should be addressed in order to alleviate sexual difficulties.

Moreover, attachment styles may develop and change over time through positive relationships and therapeutic interventions, potentially leading to changes in one’s sexual functioning and relationship dynamics.

**Disclosure of Interest:**

None Declared

